# Acidification and solar drying of manure-based digestate to produce improved fertilizing products

**DOI:** 10.1016/j.jenvman.2023.117664

**Published:** 2023-06-15

**Authors:** L. Morey, B. Fernández, L. Tey, C. Biel, A. Robles-Aguilar, E. Meers, J. Soler, R. Porta, M. Cots, V. Riau

**Affiliations:** a IRTA Institute of Agrifood Research and Technology, Torre Marimon, E08140 Caldes de Montbui, Barcelona, Spain; b EMA Depuració i Enginyería de l’Aigua, S.L. Avda. Sant Jordi, 176 Baixos, 17800, Olot, Girona, Spain; c PORGAPORCS S.L, Carrer Sant Miquel, 53, 25245, Vila-sana, Lleida, Spain; d University of Gent, Department of Green Chemistry & Technology, 9000, Gent, Belgium; e Universitat Politècnica de Catalunya, Departament de Projectes d’Enginyeria, Campus Nord, Edifici C2, 08034, Barcelona, Spain

**Keywords:** Nutrient recovery, Thermal treatment, Greenhouse solar drying, Digestate acidification, Greenhouse gas emissions, Ammonia emissions

## Abstract

The increase in energy and fertilizer consumption makes it necessary to develop sustainable alternatives for agriculture. Anaerobic digestion and digestates appeared to be suitable options. However, untreated digestates still have high water content and can increase greenhouse gas emissions during storage and land application. In this study, manure-derived digestate and solid fraction of digestate after separation were treated with a novel solar drying technology to reduce their water content, combined with acidification to reduce the gaseous emissions. The acidified digestate and acidified solid fraction of digestate recovered more nitrogen and ammonia nitrogen than their respective non-acidified products (1.5–1.3 times for TN; 14 times for TAN). Ammonia and methane emissions were reduced up to 94% and 72% respectively, compared to the non-acidified ones, while N_2_O increased more than 3 times. Dried digestate and dried acidified digestate can be labeled as NPK organic fertilizer regarding the European regulation, and the dried solid fraction and the improved dried acidified solid fraction can be labeled as N or P organic fertilizer. Moreover, plant tests showed that N concentrations in fresh lettuce leaves were within the EU limit with all products in all the cases. However, zinc concentration appeared to be a limitation in some of the products as their concentration exceeded the European legal limits.

## Highlights

• A solar drying greenhouse was used to produce organic fertilizers from digestate.
• Acidification reduced up to 94% and 72% emissions for NH3 and CH4 respectively.
• The resulting products were compared with the current fertilizer EU legislation.
• Phytotoxicity of the resulting organic fertilizers was tested with lettuce pot trials.

## 1 Introduction

The consumption of fertilizers in Europe increased 6.9% for nitrogen and 21.9% for phosphorous since 2010 (Eurostat, 2022) and it has been a tendency since the past decades (Liu et al., 2015). Therefore, the recovery of biobased fertilizers from animal manure to partially replace synthetic mineral fertilizers should be considered a key strategy to move towards a more sustainable agriculture. In this regard, anaerobic digestion is a valuable process to treat livestock manure since, apart from the generation of biogas as a renewable energy source, it allows to recycle nutrients from the derived digestate as fertilizer (Barzee et al., 2019; Horta and Carneiro, 2022), besides some other alternative valorizations (ethanol production, nutrient-enriched microalgae, or membrane concentration of specific nutrients). However, depending on the agricultural practices during their storage and land application, digestates could release volatile organic compounds and gaseous emissions, and generate water eutrophication (Battista and Bolzonella, 2019; Salamat et al., 2020). Therefore, further processing of digestates to improve their fertilizer efficiency and minimize emissions should be studied.

Different technologies have been proven to handle digestate or recover their nutrients. Mechanical separation is usually the first step, splitting digestate into concentrated and clarified fractions that are further treated afterwards. The clarified or “liquid” fraction can go through membrane separation that concentrates nitrogen and phosphorus compounds using a selective barrier (Sengupta et al., 2015) or through the stripping process to recover ammonium as ammonium sulfate (Laureni et al., 2013) and ammonium nitrate (Sigurnjak et al., 2019). The concentrated or “solid” fraction can also be treated by composting to stabilize the organic matter (Cáceres et al., 2018) or drying to reduce its water content (Angouria-Tsorochidou et al., 2022). Focusing on drying technologies, many installations have found that drying the digestate or the solid fraction of digestate is an economically viable approach because the end product would strongly reduce its volume, being more suitable for exportation due to a reduction of transportation and storage costs (Lebuf et al., 2012; Salamat et al., 2020).

Opposite to conventional thermal dryers, solar dryers have been used as a traditional method for food preservation (Jairaj et al., 2009). Moreover, in countries with high solar radiation, it is an energetically sustainable method (Ndukwu et al., 2018). In 2005, the “Institute of Heat Engineering, Warsaw University of Technology” developed the concept of solar dryers for wastewater where the energy use is considerably lower than in other drying facilities (Krawczyk and Badyda, 2011). Conventional driers (convective drying, conductive drying, fry drying) require a specific energy consumption between 700 and 1400 kWh per ton of evaporated water, while solar dryers 30–200 kWh per ton of evaporated water when they are combined with heated floors (Salamat et al., 2020). Nowadays, solar drying for sewage sludge is a reality, with companies developing solar drying treatment systems at full-scale. Recently, Battista and Bolzonella (2019) have studied the solar drying of digested slurry to recover ammonium sulfate using a solar dryer with a greenhouse configuration at the laboratory scale, promoting the use of solar energy in the treatment of waste. However, solar dryers have not been tested yet at full scale to produce organic fertilizers from manure-derived digestates.

One of the risks when drying nitrogen-rich digestates is the volatilization of nitrogen as ammonia (NH_3_) (Stambasky, 2013). In this sense, an interesting approach to reduce ammonia emissions is using acidic agents to shift the acid-base equilibrium to ammonium (NH_4_^+^) (Fangueiro et al., 2015). Prenafeta-Boldú et al. (2021) studied the combination of acidification with the solar drying of fresh pig slurry to control ammonia and greenhouse gas (GHGs) emissions on a pilot scale. Recently, Dalby et al. (2022) reported a decrease in methane emissions by 63–99% during the management of pig slurry after acidification at pH ≈ 5.5. However, there are no references to studies that measure emissions at a bigger scale and, at the same time, aim to produce fertilizers from digested manure as a valorization or post-treatment technology.

This work aimed to assess the efficiency of a nutrient recovery process of manure-based digestate that combined the acidification, solar drying, and final addition of the N-poor liquid fraction obtained after the stripping of the liquid fraction of digestate in the production of more sustainable organic fertilizers. The study focuses on reducing water content, conservation of nutrients, and reduction of greenhouse gases and acidifying emissions, followed by a pot phytotoxicity test and a comparison to the current European Fertilizers Regulation to determine the viability of the final products.

## 2 Materials and methods

### 2.1 Fertilizers production at a semi-industrial scale

**Fig. 1:**
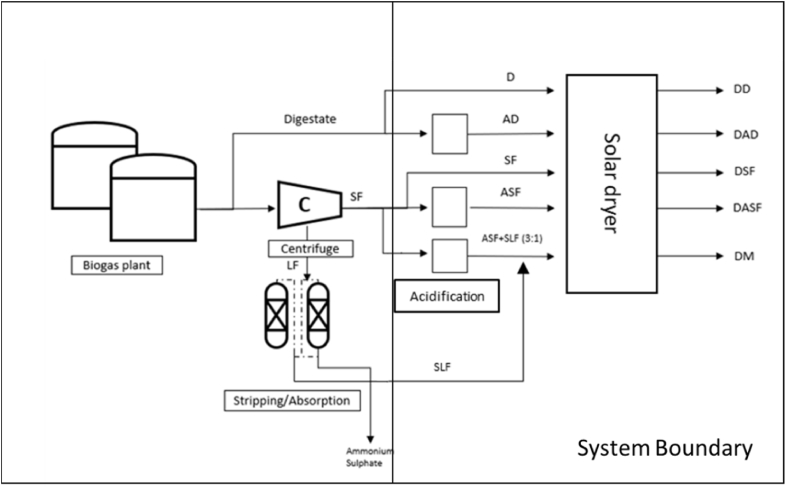
Diagram with all the digestate-derived fertilizers produced. Abbreviations: D, digestate; AD, acidified digestate; C, centrifuge; SF, solid fraction of digestate; LF, liquid fraction of digestate; ASF, acidified solid fraction; SLF, poor-nitrogen stream after LF stripping process; DD, dried digestate; DAD, dried acidified digestate; DSF, dried solid fraction; DASF, dried acidified solid fraction; DM, dried mixture of ASF and SLF.

The production of digestate-derived fertilizers (Fig. 1) was done in two periods. First, a set of four products were obtained: dried digestate (DD), dried acidified digestate (DAD), dried solid fraction of digestate (DSF), and dried acidified solid fraction of digestate (DASF). Second, after the first results, an improved trial was performed to produce a new dried acidified solid fraction of digestate (DASF2) and a dried mixture (DM) of the acidified solid fraction of digestate (ASF) with a secondary stream with a low nitrogen content (stripped liquid fraction; SLF), coming from a stripping process, in a ratio ASF:SLF of 3:1 (wet mass).

All fertilizers were produced by duplicate in a semi-industrial solar drying plant, with two greenhouse-type solar dryers (Fig. 1), beside a biogas plant (Vila-sana, Lleida, Spain) where the digestate was generated. The operational costs of the solar drying were estimated to b 4€/m^3^ of raw digestate. The biogas plant treated pig manure and agro-industrial wastes (on a yearly average, pig manure 41%, sewage sludge mixture 50%, and others 9% of total incoming daily inflow) at 37 °C with a hydraulic retention time of 50 days. The sewage sludge mixture (on a yearly average, slaughterhouse sewage sludge 30%, municipal sewage sludge 9%, dairy sewage sludge 6%, brewery sewage sludge 5%) was designed by the biogas plant operator considering that the concentration of heavy metals per each type of sludge was always below the limit defined by the Spanish regulation about the use of sewage sludge in the agricultural sector (RD 1310/1990).

The production of the digestate-derived fertilizers was performed from mid-April to mid-September (same period in years 2020 and 2021), with an ambient temperature ranging from 4.9 °C to 40.1 °C in 2020, -0.3 °C to 41.2 °C in 2021, and an annual mean solar radiation of 16.3 MJ/m^2^ (Servei Meteorològic de Catalunya, 2020). First, a concentrated stream or solid fraction (SF) was produced from digestate by a solid-to-liquid separation process (centrifugation plus a previous addition of a polyelectrolyte-type flocculant and antifoam agent).

**Fig. 2:**
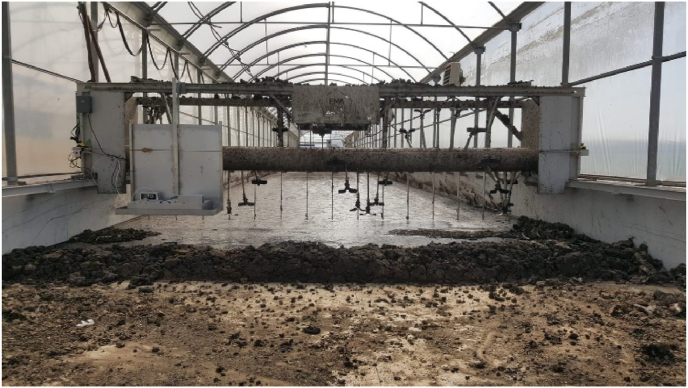
Greenhouse solar dryer and turning machine in the semi-industrial installation.

The acidification of digestate or SF was done once before drying, adding a solution of sulfuric acid (richness 50%; doses of 0.028 g-sulfuric/kg) till a pH of 5.5–6.0 according to the best available techniques to avoid ammonia emissions (Santonja et al., 2017). This process was controlled by a digital pH controller and the pump was adding sulfuric acid solution into a screw that fed the solar dryer until the desired pH was reached. Two drying lines were available; each comprised one solar dryer, one air blower, and one biofilter. Each greenhouse solar dryer had a working area of 468 m^2^ (total area of 625 m^2^; length 80 m; width 7.8 m; height 3.7 m), divided into two subareas of 234 m^2^ each (length 30 m) to dry per duplicate each product simultaneously. A turning machine (Fig. 2) distributed the material evenly and prevented crusting (maximum thickness of 40 cm). A natural airflow (mean flow of 2.8 m/s) was sucked from inside the dryer with a blower (operating 10 h/d with 100% of its maximum flow) that directed it to the biofilter (height 5.1 m; diameter 2.35 m), filled with pine bark and mature compost (ratio 10:1, in volume). The front door of the greenhouse driers remained open to promote an airflow natural convection. The drying process was operated in batch mode (4000 kg per batch for digestate or AD and 3600 kg per batch for SF or ASF).

The clarified fraction of digestate or liquid fraction (LF) after centrifugation was submitted to a stripping process (Fig. 1), followed by an absorption process to recover ammonia as ammonium sulfate (AS; richness 17%). In addition to AS, a low nitrogen concentration stream or SLF was obtained after stripping. This stream was blended with ASF, in a ratio ASF:SLF = 3:1, to produce a dried mixture (DM) to improve the nutrient (mainly nitrogen) concentration of DASF and valorize SLF or secondary stream.

During the drying process, the temperature of air and products was registered. Samples of the initial, intermediate (once per week), and final materials, as well as gaseous emissions (NH_3_, CH_4_, N_2_O), were collected (2 replicates for physic-chemical samples and GHG, and 3 replicates for NH_3_ emission samples). For the sampling, the drying area (234 m^2^) was divided into two equivalent zones (117 m^2^) where two sampling points were fixed. In these points, representative samples of the corresponding materials were taken within a 10–20 cm depth. These two sampling points per drying zone were also used to collect the gaseous emissions. Recovered total nitrogen, total ammonia nitrogen, total carbon, total phosphorus, and total potassium were compared between acid and non-acid products.

### 2.2 Gaseous emissions

Gaseous samples were taken inside the semi-industrial solar dryers using a dynamic chamber (dimensions 1030 × 530 × 250 mm; Odournet GmbH, Germany), placed directly above the material and equipped with a pump that ensured a laminar airflow (Prenafeta-Boldú et al., 2015; Torrellas et al., 2018). The temperature inside each solar dryer was registered while sampling. All samples were collected in duplicate.

The samples (30 mL) for the determination of the greenhouse gases (GHG: CH_4_, N_2_O, CO_2_) were collected in 12.5 mL vacutainer-type tubes (Labco Ltd., Buckinghamshire, UK) per duplicate. This measurement was done using a gas chromatograph (Thermo Trace 2000; Thermo Finnigan Scientific) equipped with a flame ionization detector and an electron capture detector (model 7820 A, Agilent). Results were expressed as ppm at 25 °C.

The measurement of the NH_3_ concentration was also done in situ with a portable sensor (model GX-6000, RKI Instruments; sensitivity range of 0.5–150 ppm v/v) equipped with an electrochemical detector, directly connected to the outflow of the dynamic chamber. When the sensor was not available, samples for NH_3_ determination were taken by bubbling 3 L of air (outflow of the dynamic chamber) into 20 mL acid solution (sulfuric acid 10%) tubes, which were analyzed by UV-VIS spectrometry (Hach Lange DR2800) according to the NIOSH 6015 method (Eller and Cassinelli, 1994). Results were expressed as ppm at 25 °C.

The GHG and NH_3_ emission flux (mg/m^2^h) at 25 °C was calculated by multiplying the corresponding concentration, after converting gas concentrations from ppm to mg/m^3^ by equation (1) (National Research Council, 2001) at 25 °C, by the hood internal flux (30 m3/m^2^ h). The cumulated gas emission after 21 days was calculated by the trapezoidal method of integration (Levy et al., 2017; Dalby et al., 2022).

Eq.1 ppm_NTP_ = (mg/m^3^) • (24.46/MW) • (760/P) • (T/298)

Where P, sampling site pressure (mm Hg); T, sampling site temperature (K); MW, molar weight (g/mol); NTP, normal temperature and pressure.

The measured GHGs were expressed as equivalent of CO_2_ (tCO_2_eq) using 28 mg_CO2_/mg_CH4_ and 265 mg_CO2_/mg_N2O_ as conversion factors (Myhre et al., 2013). The emissions of CO_2_ were not considered to calculate the total CO_2_ equivalent due to the livestock carbon dioxide is net-zero (Gavrilova et al., 2019).

### 2.3 Phytotoxicity assay

A pot experiment with seedlings of lettuce (variety Maravilla) was made to test the potential phytotoxicity and fertilizing effect of the dried products compared to a commercial soluble fertilizer (Agrolution). Each pot had a volume of 250 cm^3^. The substrate used in the experiment was a mixture of peat and perlite in a 1:1 ratio. The watering was done daily to keep the proper moisture content. Four aqueous extracts (ratio 1:10, in volume) of the products DD, DAD, DSF, and DASF were prepared, mixing 100 g of material and 1 L of distilled water and letting stand for 6 h before filtering. Each extract was diluted 100%, 75%, 50%, 25%, and 15% with distilled water. The pH, conductivity, TAN, and NO_3_–N were measured in each extract. After observing the high ammonium concentrations in the DAD extracts, it was decided to apply more diluted extracts only for this product: 10%, 20%, 30%, and 50% dilution. Twelve applications of 20 mL of each extract and dilution per plant were added to the corresponding pot during the growing period (n = 20). The plants were grown in a heated greenhouse (IRTA Cabrils, Spain) for 63 days avoiding temperatures below 5 °C. After harvesting, the plants' fresh weight of 10 replicates was measured, and consecutively, plants were dried at 60 °C for 72 h until constant weight. Dry biomass was ground to particles <2 mm.

### 2.4 Physic-chemical characterization of fertilizers and plant analyses

Samples of initial raw materials, as well as intermediate and final samples of dried fertilizers, were characterized. The Standard Methods (APHA, 2005) were followed to measure conductivity (method 2510); pH (method 4500-H^+^); total ammonia-nitrogen (TAN; method 4500-NH3-C), determined using a Buchi distillation unit (BUCHI Labortechnik GmbH, The Netherlands); and total solids (TS) and volatile solids (VS) (method 2540G) of all samples. The content of total carbon (TC), and total nitrogen (TN) was measured in all samples using an elemental analyzer LECO® (Leco Corporation, Michigan, USA) (ISO-13878, 1998). In addition, the products at the beginning and end of the drying process were characterized by their content of total phosphorus (TP, expressed as P_2_O_5_), total potassium (TK, expressed as K_2_O), total organic carbon (TOC), and heavy metals. The content of TP, and TK was determined by optical emission spectrometry ICP-OES (U.S. Department of Agriculture, 2018).

Plants from the growth assessment tests were subjected to microwave digestion (CEM MARS 6, USA). The nutrient content and metal concentration in the samples were analyzed by ICP-OES. The TC and TN contents were analyzed using the CN analyzer (Skalar Analytical BV, the Netherlands).

### 2.5 Data analysis and statistics

After analyzing several digestates from the biogas plant (see Supplementary Material Table S1) and comparing them with data from the literature (see Supplementary Material Table S2), it became clear that the wide variability in the characteristics of the digestate depends on the feedstock of the anaerobic digesters. The same analysis was performed with SF and SLF. Thus, given the variability of initial materials shown in Table S2, recovery indexes were calculated in this study using the mean value of each characterizing parameter for every drying batch (initial vs. final material per batch). Then, the nutrient recovery efficiency and the reduction of gaseous emissions were compared between the acidified products with their non-acidified equivalents.

One way ANOVA was used as a statistical analysis to determine the significance of the effect of acidification on the digestate and solid fraction of digestate emissions (total accumulated CH_4_, NH_3_ and N_2_O in gCO_2eq_/m^2^), with a confidence value of 95%, using IBM SPSS as statistical software (SPSS Inc., Chicago, USA).

For the plant biomass growth, Tuckey mean separation was performed via one way ANOVA both for dry weight and wet weight to compare the efficiency of the different products, using SAS 9.4 as statistical software (SAS Institute Inc. Cary, USA).

## 3 Results

### 3.1 Production performance at the semi-industrial scale

#### 3.1.1 Nutrient recovery

**Fig. 3:**
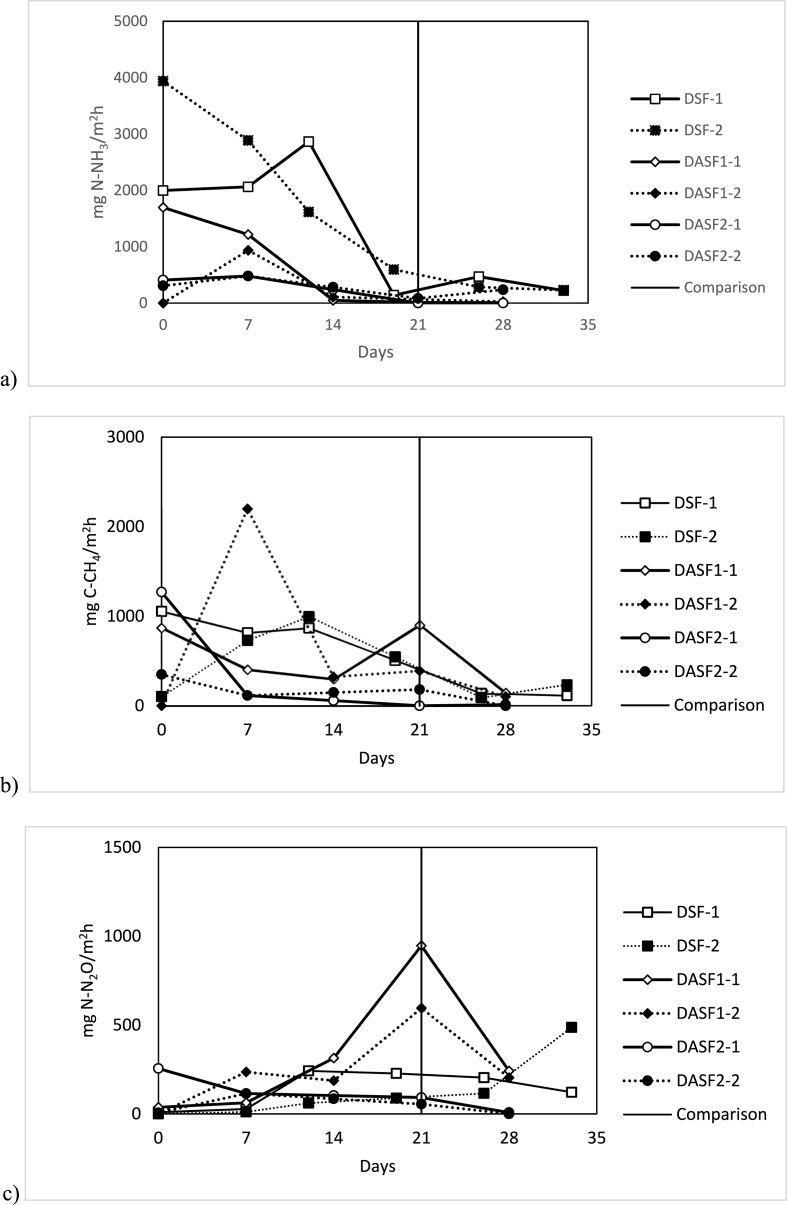
Profile of emission fluxes (mg/m^2^h), measured during the solar drying of solid fraction (SF) derived products. (a) N–NH_3_. (b) C– CH_4_. (c) N–N_2_O. Symbols: Solid line: first replicate; dashed line: second replicate; square: DSF; diamond: DASF; circle: DASF2; vertical line: 21 days were selected to calculate the total accumulated emissions per product for comparison purposes. Average values and standard deviations are represented in Table S5.

The drying process at semi-industrial scale was fulfilled between 21 and 35 days, and the emission comparison was done on the 21^st^ day to compare the emissions of all of the products (Fig. 3), depending on the products, being the drying of digestates faster than SF or ASF. This can be explained because the same amount of product occupied less volume in the case of digestate (with a 10 cm layer deposited) compared to the SF or ASF (40 cm layer along the semi-industrial dryer). All the products were dried to attain a TS of 90%, except for the mixture that was dried until a lower TS content (close to 50%) to be easily applied in fields and fulfilled the farmers' requirement. The average chemical composition of the raw materials (Digestate, SF, and SLF) and dried products are shown in Table 1.

**Table 1:** Physic-chemical characterization of digestate-derived products. Abbreviations: % wm, wet mass base; % TS, dry mass base; n.d., not determined; D, raw digestate; DD, dried digestate; DSF, dried solid fraction of digestate; DAD, dried acidified digestate; DASF, DASF2, dried acidified solid fraction of digestate; DM, dried mixture of ASF and SLF. Note: *mean values (see Supplementary Material Tables S1, S2, and S3).

Parameter	units	Raw materials			Semi-industrial scale				Improvement trial	
		D*	SF*	SLF*	DD*	DSF*	DAD*	DASF*	DM*	DASF2*
Conductivity	mS/cm	27	n.d.	n.d.	4.6	1.8	20.4	2.9	6.0	6.6
pH	–	8.3	n.d.	n.d.	7.8	7.5	5.7	8.0	6.4	7.9
TS	%wm	6.5	23	2.9	90	91	89	85	47	83
VS	%TS	63	62	63	60	60	59	61	39	57
P_2_O_5_	%TS	2.8	2.7	n.d.	6.3	6.9	3.8	6.9	6.1	5.1
K_2_O	%TS	1.4	0.3	n.d.	1.6	0.4	2.5	0.5	0.4	0.5
TC/TN		0.6	6.9	n.d.	6.7	10	3.8	8.4	8.4	7.8
TC	%TS	29	38	n.d.	33	34	25	32	25	25
TOC	%TC	–	n.d.	n.d.	100	100	100	100	67	100
TN	%TS	48	5.5	12	4.9	3.4	6.5	3.8	6.2	3.2
TAN	%TS	8.1	2.3	7.4	0.3	0.3	3.5	3.3	0.8	1.1
Cd	mg/kgTS	<0.5	n.d.	n.d.	<0.5	<0.5	<0.5	<0.5	<0.5	<0.5
Cu	mg/kgTS	167	n.d.	n.d.	149	152	95	173	134	115
Cr (VI)	mg/kgTS	<0.5	n.d.	n.d.	<0.5	<0.5	<0.5	<0.5	n.d.	n.d.
Hg	mg/kgTS	<0.4	n.d.	n.d.	<0.4	<0.4	<0.4	<0.4	<0.4	<0.4
Ni	mg/kgTS	19	n.d.	n.d.	16	12	17	13	21	16
Pb	mg/kgTS	7.2	n.d.	n.d.	7.7	6.9	<5	7.4	8.1	6.9
Zn	mg/kgTS	884	n.d.	n.d.	738	780	512	891	816	663

Acidified products (DAD and DASF) recovered more TN and TAN than the non-acidified products (DD and DSF): 1.5 and 1.3 times, respectively, for TN; 14 times each one for TAN. Concerning potassium, both acidified products recovered more potassium than the non-acidified ones (1.7 times for DAD and 1.4 times for DASF). The recovered TP and TC of DAD production were lower than in its non-acidified relative product (0.68 and 0.86 times, respectively). On the other hand, the recovery of TP and TC of DASF production was higher than in the DSF production (1.13 and 1.07 times, respectively).

#### 3.1.2 Gaseous emissions

Emissions of ammonia and GHGs were measured for each fertilizer production, under batch mode, in the semi-industrial driers. Table 2 summarizes the total cumulative emissions (NH_3_, CH_4,_ and N_2_O).

**Table 2:** Total cumulative emission of GHGs and ammonia (NH_3_) after 21 days. Note: Sum of net CH_4_, N_2_O, expressed in CO_2_ eq units/m^2^. (*) Comparison between DSF and DASF2 or DM. Abbreviations: DD, dried digestate; DSF, dried concentrated fraction of digestate; DAD, dried and acidified digestate; DASF, dried and acidified solid fraction of digestate 2020; DASF2, dried and acidified solid fraction of digestate 2021; DM, dried mixture of ASF:SLF; non-AP, non-acidified product. Standard deviation can be found in supplementary material (Table S4).

Total emitted	Units	DD	DAD	DSF	DASF	*DASF2	*DM
CH_4_	kg CO_2_ eq/m^2^	2.4	1.08	11	12	4.8	5.7
CH_4_	p value	0.383		0.779		<0.001	0.122
N_2_O	kg CO_2_ eq/m^2^	8.2	59	35	124	17	25
N_2_O	p value	0.007		0.005		0.218	0.524
NH_3_	g NH_3_/m^2^	389	25	1169	327	223	235
NH_3_	p value	<0.001		<0.001		<0.001	<0.001

Ammonia emissions decreased during the drying of acidified products (Fig. 3). The total cumulative emission of NH_3_ were 25 and 327 g-NH_3_/m^2^ for DAD and DASF, respectively, while these emissions for the corresponding non-acidified products were 389 and 1169 g-NH_3_/m^2^ for DD and DSF, respectively. This means that acidification reduced significatively the total cumulative NH_3_ emission by 94% drying digestate and 72% drying the solid fraction of digestate (SF).

Regarding DASF compared to DSF, acidification caused an increment of N_2_O and CH_4_ emissions, 251% (p < 0.05), and 5.8% (p > 0.05) respectively. Concerning DAD compared to DD, there is an increase in N_2_O, 620% (p < 0.05), and a reduction of CH_4_ of 54% (p > 0.05).

#### 3.1.3 Improved production of dried fertilizing products

Based on previous results, the turning machine was modified to improve the aeration, by installing perpendicular flippers to increase the removal of the crust, and the sulfuric acid addition was improved by changing the dosing point, which allowed a better pH measurement. Within these changes, a second batch for drying ASF was performed, producing DASF2 (Table 1, Table 2). Based on a comparison with DSF, a clear reduction of all emissions was shown, including N_2_O and CH_4_. As can be seen in Fig. 3, the effect of acidification is clearly improved, not only related to the reduction of emissions but also reducing the deviation between replicates. For DASF2, emission was reduced significatively by 81% and 57% for NH_3_ and CH_4_, and a reduction (p > 0.05) of 53% for N_2_O, compared to DSF.

Another improved approach was the mixture of the ASF with a waste stream coming from the stripping plant from the anaerobic digestion facility (SLF). The stream ASF was the best candidate to produce the mixture as it contained the greatest TP content and showed a clear decrement in NH_3_ emissions while drying. The idea of producing the mixture was to enhance the fertilizing value of the acidified solid fractions (ASF) by using the SLF as an additional nitrogen source. In addition, a secondary objective was to valorize the high volume of SLF, that remained after the stripping of LF. Therefore, ASF:SLF was blended in a 3:1 ratio and dried. As a result, the TN content of the dried mixture was higher than the TN content of the DASF (Table 1); however, the conductivity of the mixture also increased as the SLF still had a high concentration of salts. Regarding gaseous emissions, compared to DSF, the NH_3_ emission was reduced by 80% (p < 0.05), 28% for N_2_O (p > 0.05), and 50% (p > 0.05) for CH_4_.

### 3.2 Phytotoxicity effect of the recovered products

A plant growth test was performed with extracts of the recovered products (not the mixtures) to observe possible phytotoxic effects in the juvenile stage of lettuce. The extracts applied had different values of pH, EC, and NH_4_^+^ (Table 3), which significantly affected the lettuce's fresh and dry weight (Table 4).

**Table 3:** pH, EC, and NH_4_^+^ concentrations measured in the extracts. Abbreviations: n.a., not available. Standard deviation can be found in supplementary materials (Table S6).

Extracts															
Treatment	pH					EC (mS/cm)					NH_4_^+^ (mg/L)				
Dilution	15	25	50	75	100	15	25	50	75	100	15	25	50	75	100
DD	n.a.	7.8	7.8	7.8	7.8	n.a.	2.2	3.2	4.4	5.7	n.a.	22	34.5	49	64
DSF	n.a.	8	7.9	7.9	7.9	n.a.	1.4	1.7	2.1	2.5	n.a.	24	42.0	63	83
DASF	n.a.	8	7.9	7.9	8.0	n.a.	1.5	1.7	2.0	2.5	n.a.	9.4	19.0	28	40
Dilution	10	20	30	50	/	10	20	30	50	/	10	20	30	50	/
DAD	7.4	7.1	6.9	6.6	n.a.	3.1	5.3	7.3	11	n.a.	216	256	282	284	n.a.

**Table 4:** Lettuce fresh and dry weight (g plant^−1^), total nitrogen (TN), total carbon (TC), and leaves Zn concentrations. Note: *Plants were dead. Standard deviation can be found in supplementary materials (Table S7).

Treatment	DD				DAD				DSF				DASF			
Dilution	25	50	75	100	10	20	30	50	25	50	75	100	25	50	75	100
g plant ^−1^																
Fresh weight	2.5 (bcde)	3.9 (a)	3.5 (ab)	3.3 (abc)	2.0 (de)	1.9 (de)	1.7 (d)	†*	2.9 (abcd)	2.3 (cde)	3.4 (ab)	3.5 (ab)	1.6 (e)	2.1 (de)	1.6 (e)	2.3 (cde)
Dry weight	0.25 (abcd)	0.3 (a)	0.29 (ab)	0.26 (abc)	0.2 (bcd)	0.16 (d)	0.18 (cd)	†	0.3 (abc)	0.2 (abcd)	0.25 (abcd)	0.26 (abc)	0.2 (abcd)	0.23 (abcd)	0.2 (cd)	0.26 (abc)
% TS																
TN	3.0	3.2	3.5	3.9	5.5	6.2	6.3	†	2.7	3.2	4.1	4.3	2.9	3.0	3.4	3.4
C	38	37	37	35	37	37	37	†	35	37	38	37	39	37	38	38
mg/kgTS																
Zn	128	161	109	102	129	154	121	†	56	75	171	165	72	108	107	102

The maximum concentration applied for DAD in this growth test was 50%. Still, the ammonium content in the extracts was much higher in this treatment than with the other products. Consequently, N concentrations analyzed in plants treated with DAD (>6 gN/100 gDW-plant) (Table 4) were in the high or toxic range of N in plant tissue as defined by Marschner (2011).

The dose applied did not have a significant effect on the plant biomass within each treatment (p value < 0.0001); however, non-acidified products led to higher biomass in general than the acidified counterparts. Furthermore, plants treated with >30% extracts of DAD died, indicating a growth inhibition due to the high EC and NH_4_^+^ concentrations found in this treatment.

After observing Zn concentrations higher than regulation limits (Zn > 800 mg/kg) in the chemical analyses of the DASF, the concentrations of metals in plant tissue were analyzed, to measure if they were within the average values for lettuce. The results showed no significant differences in Zn uptake in plants treated with DASF compared to plants treated with the non-acidified DSF. Despite the Zn concentrations found in edible tissues of lettuce (Table 4) being higher than the expected value (Li et al., 2016), the EU regulation does not include a plant tissue limit concentration approach. Other elements analyzed in the tissue of lettuce were within the normal range, and elements such as Cd, Co, Cr, Cu, Ni, and Pb were less than the detection limit.

## 4 Discussion

### 4.1 Solar drying combined with acidification improved the efficiency and sustainability of the process at the pilot scale

Acidified products DAD and DASF recovered 1.5 and 1.3 times more TN and 14 and 14 times more TAN than the non-acidified products, respectively. The obtained values of nutrient recovery after acidification are higher than the ones reported previously by Liu et al. (2019), who recovered up to 6.2 times more TAN in a thermal process with acidified digestate at pH 6.5 (1 point higher than in this study) than the same digestate without acidification. This increment could be explained by the microbial activity regarding ammonification and/or the hydrolysis of organic nitrogen (Moure Abelenda et al., 2021), and as it appeared to be, the lower the pH, the higher the N recovery, especially for total ammonia nitrogen, as ammonium fraction (ionic form) is higher than ammonia (non-ionic/volatile form).

Ammonia emissions dramatically decreased during the drying of acidified products, 94% and 72% for DAD and DASF respectively, and a major decrease of 81% and 80% for DASF2 and DM compared to DSF. This is in line with the data reported by Wagner et al. (2021), which determined a decrease of 89–96% in ammonia emissions during the field application of acidified pig slurry (pH 6), compared to the non-acidified one. Eihe et al. (2019) obtained a reduction of up to 90% of NH_3_ emission with acidified digestate (pH 6.5) during its application to the field, compared to the non-acidified digestate.

Despite the turning machine that removed the upper crust during the drying for the solid fraction products, total or partial nitrification conditions could be attained in the lower part of the material. The peak of N_2_O emissions was placed when the TS content of the composite sample was> 30%. Moreover, acidification increased the available ammonia nitrogen to be transformed by nitrogen oxidizing bacteria (Cytryn et al., 2012) to produce N_2_O, NO_2_ or NO_3_ than in the non-acidified product. Nevertheless, after the improvements were done in the turning machine and the acidification, N_2_O emission was reduced compared to the previous system (the reduction of N_2_O emission was 53%) due to the reduction of crust in the surface. In the case of digestate drying, the formation of the crust, which was not removed, and the enhance of nitrogen oxidizing bacteria due to acidification could explain the high emission levels of N_2_O.

In addition, acidification is supposed to inhibit methanogenesis by decreasing the activity of methanogenic bacteria (Pantelopoulos and Aronsson, 2021). However, the mixture of solid fraction with acid did not differ widely from de non-acidified. This can be related to a non-optimized acidification procedure because after the system was improved, methane emissions decreased compared to the non-acidified product (Table 2). When the acidification system was improved for DASF2, methane emission was reduced considerably, up to 57%.

There are few references regarding digestate acidification, but some regarding the acidification of fresh manure. Miranda et al. (2021) presented a 98% reduction in CH_4_ emissions by acidifying weekly cattle slurry to maintain a pH of 5.5 during storage. Im et al. (2020) demonstrated a 70% reduction of methane emissions during pig slurry storage acidifying to pH 6.5. Pantelopoulos and Aronsson (2021) showed a 50% reduction with a liquid fraction of pig slurry acidified to 5.9. All of them with similar methane reduction values as those obtained by the acidification of digestate in this study (73%).

### 4.2 Biobased products obtained as candidates for the European fertilizer legislation and RENURE criteria

These products were compared with the European criteria to determine their potential application as organic fertilizers, following Regulation 2019/1009 of the European Parliament and of the Council (Regulation EU, 2019/1009), describing the requirements and the limitations on the application of organic fertilizers. In general, NPK solid organic fertilizers must have a concentration of TN > 1% TS, P_2_O_5_ > 1% TS, K_2_O > 1% TS, NPK >4% TS, and TOC >15% TS. For a solid organic fertilizer declaring only a primary nutrient, the required concentrations are TN > 2.5% TS, or P_2_O_5_ > 2% TS, or K_2_O > 2% TS.

According to the EU regulation, regarding nutrients (NPK) criteria, only DD and DAD, with nutrient concentrations higher than the minimum established by the legislation, are suitable to get the CE label to be marketed in the European Union. Each country's regulation will determine the limitations to be used in the field. Considering only N or P fertilizer criteria, all the products were appropriate to be applied as fertilizers.

However, DASF and DM would be rejected due to their concentration of Zn which was above the threshold of 800 mg/kg. This is explained by the high concentrations of Zn found in the pig slurry used as a major substrate in the anaerobic digestion plant of this study: zinc is normally used as an additive to stimulate animal growth and prevent diseases (Alburquerque et al., 2012). The presence of heavy metals in digestate is associated more to the manure than the agricultural wastes, related to the supplements for commercial feedstuff to promote optimum nutrient supply and growth (Demirel et al., 2013).

Also, the dried products were compared with future guidelines for recovered nitrogen from manure (RENURE) products that define the quality and/or handling rules that a processed manure material should comply to be classified as a “*substance fully or* partially derived from livestock manure through processing that can be used in areas with water pollution by nitrogen following otherwise identical provisions applied to nitrogen containing chemical fertilizers as defined in the Nitrates Directive (91/676/EEC), while ensuring the achievement of the Nitrates Directive's objective and *providing adequate agronomic benefits to enhance plant growth*” (Huygens et al., 2020). It is necessary to fulfill one of the following criteria: (i) TOC:TN 0 ≤ p ≤ 3 or (ii).

TAN:TN > 90%, where p is the evaluated product. Moreover, copper and zinc concentrations must be < 300 and < 800 mg/kg, respectively. In agreement with the RENURE criteria, only DASF was close to fulfill the requirements with a TAN: TN ratio of 89%. However, Zinc concentration was higher than the established limit of 800 mg/kg, as well. Reuland et al. (2021) analyzed 2800 data from unpublished and literature digestate and liquid fraction analyses for RENURE criteria, and the results showed that liquid fraction is better suited to RENURE criteria than digestate, with compliance between 43 and 58%.

The plant tests showed that N concentrations in lettuce fresh leaves were within the EU limit (<3000 mg/kg fresh weight) with all products in all the cases, as the European Union establishes maximum permissible levels from 4000 to 5000 mg N–NO_3_^-^/kg fresh weight for the winter season (Commission Regulation 1258/2011). However, the N concentrations (>6 gN/100 g TS plant) (Table 4) analyzed in lettuce plants treated with DAD were in the high/toxic range of N in plant tissue as defined by Marschner (2011). Interestingly, even though Zn concentration in DASF was higher than law limits (>800 mg/kg), no significant differences in Zn uptake were observed compared to plants treated with the non-acidified DSF in the higher doses. Also, Zn concentrations found in edible tissues of lettuce were higher than the expected value of 7.9 mg/kg TS (Li et al., 2016), but no limit is established in the regulation.

Although more studies need to be conducted to study the full growing cycle of lettuces, we can preliminarily advance that all concentrations of DD, DSF and DASF can be applied for growing lettuces, as the dose applied did not have an effect on the plant biomass and the concentration of heavy metals and NO3 are below the regulation limits. However, for DAD, concentrations higher than 30% can have a negative effect on the plants.

## 5 Conclusions

DASF and DAD recovered 1.3 and 1.5 times more total nitrogen than the non-acidified DSF and DD counterparts and 14 times more ammonia. Moreover, the acidified products reduced the ammonia emissions by up to 94% and 72% for DAD and DASF, compared to the non-acidified ones. On the other hand, N_2_O emissions increased 620% and 251% for DAD and DASF, compared to their non-acidified relatives, DD and DAD fit the European regulation of fertilizers to be labeled as solid organic NPK fertilizers, and DSF, DASF, and DM could be labeled as Nitrogen or Phosphorus solid organic fertilizers. DASF and DM had a concentration of Zn superior to that regulation's established limit, but no significant differences in Zn concentration appeared in the plant leaves. Moreover, the dose of application did not have a significant effect on plant biomass. Finally, plant tests showed that N concentrations in fresh lettuce leaves were within the EU limit with all products in all the cases.

## Credit author statement

L. Morey: Methodology, Validation, Formal analysis, Investigation, Writing – original draft, Writing – review & editing. B. Fernández: Conceptualization, Methodology, Validation, Investigation, Writing – review & editing, Supervision. L. Tey: Formal analysis, Investigation. C. Biel: Conceptualization, Methodology, Formal analysis, Investigation, Writing – review & editing. A. Robles-Aguilar: Validation, Formal analysis, Investigation, Writing – review & editing. E. Meers: Writing – review & editing, Supervision, Funding acquisition. J. Soler: Resources. R. Porta: Resources. M. Cots: Resources. V. Riau: Conceptualization, Methodology, Validation, Investigation, Writing – review & editing, Supervision, Project administration, Funding acquisition

## Declaration of competing interest

The authors declare that they have no known competing financial interests or personal relationships that could have appeared to influence the work reported in this paper.

## Data Availability

Data will be available in a public repository
