# Supplementary material for: Acidification and solar drying of manure-based digestate to produce improved fertilizing products
Source: J Environ Manage. 2023 Jun 15;336:117664. doi: 10.1016/j.jenvman.2023.117664 (PMC10060120; doi:10.1016/j.jenvman.2023.117664)
Supplement: Multimedia component 1 [file mmc1.docx]

Supplementary material

Table S1. Characteristics of digestates (D), concentrated fractions of digestates (SF), clarified fraction of digestates (LF), sampled in 2 years of experimentation.

|  | TC | TN | N-NH_4_^+^ | TS | VS | TP | TK |
| --- | --- | --- | --- | --- | --- | --- | --- |
| Dry weight | % | % | % | % | % | % | % |
| Digestate D | 33 | 10 | 6.9 | 5.3 | 64 | 2.9 | 1.4 |
|  | - | 22 | 14 | 2.9 | 65 | 2.7 | 1.3 |
|  | 19 | 76 | 7.2 | 6.9 | 64 | - | - |
|  | 32 | 90 | 8.1 | 6.5 | 65 | - | - |
|  | 33 | 91 | 7.3 | 6.9 | 64 | - | - |
|  | 34 | 86 | 7.1 | 7.1 | 65 | - | - |
|  | 34 | 78 | 7.1 | 7.1 | 67 | - | - |
|  | 37 | 86 | 7.8 | 6.6 | 62 | - | - |
|  | 35 | 87 | 7.4 | 6.8 | 64 | - | - |
|  | 35 | 87 | 7.4 | 6.8 | 64 | - | - |
|  | 34 | 68 | 6.7 | 7.7 | 65 | - | - |
|  | 34 | 74 | 7.1 | 7.1 | 62 | - | - |
|  | 30 | 11 | 7.5 | 7.0 | 60 | - | - |
|  | 30 | 19 | 13 | 4.0 | 92 | - | - |
|  | 29 | 11 | 7.5 | 7.1 | 54 | - | - |
|  | 24 | 15 | 10 | 5.2 | 58 | - | - |
|  | 29 | 11 | 7.3 | 7.3 | 56 | - | - |
|  | 28 | 38 | 7.8 | 6.9 | 58 | - | - |
|  | 29 | 37 | 7.2 | 7.4 | 59 | - | - |
|  | 29 | 10 | 7.0 | 7.4 | 59 | - | - |
|  | 28 | 10 | 7.1 | 7.3 | 56 | - | - |
| Average D | 29 | 48 | 8.1 | - | 63 | 6.4 | 1.6 |
| Std.dev. | 7.7 | 34 | 1.9 | - | 7.5 | 0.3 | 0.1 |
| Liquid fraction of digestate  LF | 34 | 38 | 32 | 1.0 | 66 | - | - |
|  | 31 | 17 | 13 | 3.5 | 56 | - | - |
|  | 32 | 24 | 20 | 2.3 | 61 | - | - |
|  | 31 | 19 | 19 | 3.3 | 63 | - | - |
|  | 48 | 59 | 43 | 0.9 | 65 | - | - |
| Average  LF | 35 | 32 | 25 | - | 62 | - | - |
| Std.dev. | 6.4 | 16 | 11 | - | 3.4 | - | - |
| Solid fraction of digestate SF | 34 | 5.2 | 1.9 | 24 | 58 | 2.7 | 0.3 |
|  | 31 | 5.9 | 1.8 | 25 | 59 | 2.7 | 0.4 |
|  | 58 | 6.2 | 4.2 | 14 | 57 | - | - |
|  | 59 | 5.8 | 2.0 | 20 | 99 | - | - |
|  | 29 | 5.2 | 1.9 | 29 | 56 | - | - |
|  | 31 | 5.1 | 2.1 | 24 | 57 | - | - |
|  | 33 | 5.1 | 2.2 | 25 | 57 | - | - |
|  | 33 | 5.1 | 2.2 | 25 | 57 | - | - |
| Average SF | 38 | 5.4 | 2.3 | - | 62 | 2.7 | 0.3 |
| Std.dev. | 11 | 0.4 | 0.8 | - | 14 | 0.0 | 0.1 |

Table S2. Comparison with literature. Abbreviations: LF (liquid fraction); SF (solid fraction); n.a. (not available).

| Material | Influent of the biogas plant | TS (%) | VS (%TS) | TC (%TS) | TN (%TS) | TAN (%TS) | TP (%TS) | TK (%TS) | Reference |
| --- | --- | --- | --- | --- | --- | --- | --- | --- | --- |
| D | 41% pig manure - 59% agro-industrial wastes | 6.5 ±1.2 | 63±7.5 | 29±7.7 | 48±34 | 8.1±1.9 | 2.8±0.1 | 1.4±0.1 | this study |
| LF | 41% pig manure - 59% agro-industrial wastes | 2.6±1.0 | 62±3.4 | 35±6.4 | 32±16 | 25±11 | n.a. | n.a. | this study |
| SF | 41% pig manure - 59% agro-industrial wastes | 23±4.1 | 62±14 | 38±11 | 5.5±0.4 | 2.3±0.7 | 2.7±0.1 | 0.3±0.1 | this study |
| D | Agricultural wastes+ cattle manure + poultry manure | 4.3 | 50 | n.a. | 11 | 6.4 | n.a. | n.a. | Torrisi et al., 2021 |
| D | Pig manure + energetic crops | 4.4 | 70 | 40 | 12 | 8.8 | 1.0 | 2.7 | Jimenez et al., 2020 |
| D | Average of pig slurry, cow slurry and energetic crops | 6.1 | n.a. | n.a. | 8.1 | 4.9 | 1.6 | n.a. | Tambone et al., 2017 |
| LF | Sugar beet pulp | 3.8 | 86 | 15 | 8.5 | 4.9 | 0.1 | 5.6 | Chuda & Zieminski, 2021 |
| LF | Cattle manure | 8.8 | 39 | n.a. | 8.4 | 4.4 | 4.3 | 11 | Valentinuzzi et al., 2020 |
| LF | Pig slurry, cow slurry and energetic crops | 4.5 | n.a. | n.a. | 9.8 | 6.0 | 1.6 | n.a. | Tambone et al., 2017 |
| SF | Pig slurry, cow slurry and energetic crops | 21 | n.a. | n.a. | 2.9 | 1.0 | 1.3 | n.a. | Tambone et al., 2017 |
| SF | Wastewater treatment sludge | 22 | 52 | 27 | 4.0 | 1.1 | 2.0 | 0.2 | Jimenez et al., 2020 |

Table S3. Average and standard deviation of the physic-chemical characterization of the organic fertilizers. n = 2.

|  |  | Semi-industrial scale | | | | Improvement trial | |
| --- | --- | --- | --- | --- | --- | --- | --- |
| Parameter | units | DD | DSF | DAD | DASF | DM | DASF2 |
| Conductivity | mS/cm | 4.6 ± 0.1 | 1.8 ± 0.7 | 20 ± 3.1 | 2.9 ± 0.2 | 6.0 ± 0.6 | 6.6 ± 1.7 |
| pH | - | 7.8 ± 0.1 | 7.5 ± 0.1 | 5.7 ± 0.2 | 8.0 ± 0.1 | 6.4 ± 0.1 | 7.9 ± 0.5 |
| TS | %wm | 90 ± 2.1 | 91 ± 4.4 | 89 ± 0.6 | 85 ± 2.2 | 47 ± 1.3 | 83 ± 0.6 |
| VS | %TS | 60 ± 2.5 | 60 ± 2.5 | 59 ± 1.1 | 61 ± 1.3 | 39 ± 1.1 | 57 ± 0.2 |
| TC | %TS | 33 ± 0.8 | 34 ± 0.1 | 25 ± 3.0 | 32 ± 3.4 | 25 ± 0.8 | 25 ± 2.4 |
| TN | %TS | 4.9 ± 0.1 | 3.4 ± 0.1 | 6.5 ± 1.4 | 3.8 ± 0.1 | 6.2 ± 0.0 | 3.2 ± 0.1 |
| TAN | %TS | 0.3 ± 0.0 | 0.3 ± 0.0 | 3.5 ± 0.4 | 3.3 ± 0.0 | 0.8 ± 0.1 | 1.1 ± 0.0 |

Table S4. Average and standard deviation of the total emitted GHG and NH_3_. n = 2.

| Total emitted | Units | DD | DAD | DSF | DASF | *DASF2 | *DM |
| --- | --- | --- | --- | --- | --- | --- | --- |
| CH_4_ | kg CO_2_ eq/m^2^ | 2.4 ± 0.2 | 1.08 ± 0.5 | 11 ± 1.4 | 12 ± 4.3 | 4.8 ± 1.0 | 5.7 ± 1.3 |
| CH_4_ | p value | 0.383 | | 0.779 | | <0.001 | 0.122 |
| N_2_O | kg CO_2_ eq/m^2^ | 8.2 ± 3.7 | 59 ± 25 | 35 ± 25 | 124 ± 33 | 17 ± 11 | 25 ± 15 |
| N_2_O | p value | 0.007 | | 0.005 | | 0.218 | 0.524 |
| NH_3_ | g NH_3_/m^2^ | 389 ± 0.0 | 25 ± 0.0 | 1169 ± 0.0 | 327 ± 0.0 | 223 ± 0.0 | 235 ± 0.0 |
| NH_3_ | p value | <0.001 | | <0.001 | | <0.001 | <0.001 |

Table S5. Profile of emission fluxes (mg/m^2^h) measured during the solar drying of solid fraction derived products (average and standard deviation). Data corresponding to Figure 3. Abbreviations: DSF-1, dried solid fraction sampling point 1; DSF-2, dried solid fraction sampling point 2; DASF1-1, dried acidified solid fraction sampling point 1; DASF1-2, dried acidified solid fraction sampling point 2; DASF2-1, improved dried acidified solid fraction sampling point 1; DASF2-2, improved dried acidified solid fraction sampling point 2. Note: n = 2, * Samples measured with sensor, 1 value per spot.

|  | mgN-NH_3_/m^2^h | | | | | |
| --- | --- | --- | --- | --- | --- | --- |
| Day | DSF-1 | DSF-2 | DASF1-1 | DASF1-2 | DASF2-1* | DASF2-2* |
| 0 | 1997 ± 349 | 3936 ± 822 | 1696 ± 328 | 937 ± 332 | 408 | 307 |
| 7 | 2062 ± 641 | 2885 ± 438 | 1216 ± 31 | 108 ± 44 | 479 | 479 |
| 14 | 2864 ± 238 | 1615 ± 326 | 48 ± 33 | 65 ± 12 | 240 | 281 |
| 21 | 139 ± 82 | 594 ± 39 | 11 ± 11 | 26 ± 7.4 | 0 | 83 |
|  |  |  |  |  |  |  |
|  | mgC-CH_4_/m^2^h | | | | | |
| Day | DSF-1 | DSF-2 | DASF1-1 | DASF1-2 | DASF2-1 | DASF2-2 |
| 0 | 1054 ± 1052 | 102 ± 32 | 868 ± 743 | 2197 ± 497 | 1270 ± 340 | 349 ± 67 |
| 7 | 816 ± 257 | 731 ± 70 | 404 ± 48 | 322 ± 2.1 | 114 ± 75 | 114 ± 25 |
| 14 | 869 ± 481 | 997 ± 705 | 300 ± 267 | 389 ± 3.1 | 57 ± 1.0 | 148 ± 23 |
| 21 | 507 ± 7.3 | 545 ± 6.3 | 899 ± 288 | 102 ± 32 | 13 ± 11 | 181 ± 7.3 |
|  |  |  |  |  |  |  |
|  | mgN-N_2_O/m^2^h | | | | | |
| Day | DSF-1 | DSF-2 | DASF1-1 | DASF1-2 | DASF2-1 | DASF2-2 |
| 0 | 9 ± 11 | 0.0 ± 0.0 | 37 ± 33 | 236 ± 18 | 255 ± 165 | 6.0 ± 3.2 |
| 7 | 27 ± 22 | 11 ± 1.7 | 63 ± 17 | 187 ± 21 | 115 ± 37 | 115 ± 3.6 |
| 14 | 242 ± 307 | 60 ± 144 | 314 ± 287 | 595 ± 12 | 103 ± 94 | 85 ± 8.4 |
| 21 | 228 ± 4.1 | 89 ± 9.0 | 947 ± 16 | 204 ± 28 | 92 ± 6.1 | 55 ± 3.9 |

Table S6. Average and standard deviation of pH, EC, and NH_4_^+^ concentrations measured in the extracts. n = 6.

| Extracts | | | | | | | | | | | | | | | |
| --- | --- | --- | --- | --- | --- | --- | --- | --- | --- | --- | --- | --- | --- | --- | --- |
| Treatment | pH | | | | | EC (mS/cm) | | | | | NH_4_^+^ (mg/L) | | | | |
| Dilution | 15 | 25 | 50 | 75 | 100 | 15 | 25 | 50 | 75 | 100 | 15 | 25 | 50 | 75 | 100 |
| DD | n.a. | 7.8 ± 0.4 | 7.8 ± 0.4 | 7.8 ± 0.4 | 7.8 ± 0.4 | n.a. | 2.2 ± 0.3 | 3.2 ± 0.3 | 4.4 ± 0.2 | 5.7 ± 0.3 | n.a. | 21.9 ± 12 | 34.5 ± 12 | 49.3 ± 14 | 64.1 ± 20 |
| DSF | n.a. | 8.0 ± 0.1 | 7.9 ± 0.1 | 7.9 ± 0.2 | 7.9± 0.2 | n.a. | 1.4 ± 0.1 | 1.7 ± 0.1 | 2.1 ± 0.1 | 2.5 ± 0.2 | n.a. | 24.0 ± 11 | 42.0 ± 10 | 63.0 ± 20 | 83.4 ± 26 |
| DASF | n.a. | 8.0 ± 0.1 | 7.9 ± 0.1 | 7.9 ± 0.1 | 8.0 ± 0.1 | n.a. | 1.5 ± 0.3 | 1.7 ± 0.1 | 2.0 ± 0.1 | 2.5 ± 0.2 | n.a. | 9.4 ± 8.4 | 19.0 ± 8.2 | 27.9 ± 18 | 39.6 ± 21 |
| Dilution | 10 | 20 | 30 | 50 | / | 10 | 20 | 30 | 50 | / | 10 | 20 | 30 | 50 | / |
| DAD | 7.4 ± 0.4 | 7.1 ± 0.3 | 6.9 ± 0.3 | 6.6 ± 0.2 | n.a. | 3.1 ± 0.5 | 5.3 ± 0.5 | 7.3 ± 0.6 | 11 ± 0.7 | n.a. | 216 ± 143 | 256 ± 155 | 282 ± 109 | 284 ± 104 | n.a. |

Table S7. Lettuce fresh and dry weight (g plant^-1^) average and standard deviation, total nitrogen (TN), total carbon (TC), and leaves Zn concentrations (n=1; composed from X samples). Note: *Plants were dead. n = 10 (fresh and dry weight).

| Treatment | DD | | | | DAD | | | | DSF | | | | DASF | | | |
| --- | --- | --- | --- | --- | --- | --- | --- | --- | --- | --- | --- | --- | --- | --- | --- | --- |
| Dilution | 25 | 50 | 75 | 100 | 10 | 20 | 30 | 50 | 25 | 50 | 75 | 100 | 25 | 50 | 75 | 100 |
| g plant ^-1^ | | | | | | | | | | | | | | | | |
| Fresh weight | 2.5 ± 0.8 (bcde) | 3.9 ± 0.8 (a) | 3.5 ± 0.8 (ab) | 3.3 ± 1.0 (abc) | 2.0 ± 0.5 (de) | 1.9 ± 0.4 (de) | 1.7 ± 0.4 (d) | †* | 2.9 ± 0.9 (abcd) | 2.3 ± 0.5 (cde) | 3.4 ± 0.6 (ab) | 3.5 ± 0.9 (ab) | 1.6 ± 0.5 (e) | 2.1 ± 0.5(de) | 1.6 ± 0.6 (e) | 2.3 ± 0.7 (cde) |
| Dry weight | 0.25 ± 0.1 (abcd) | 0.3 ± 0.1 (a) | 0.29 ± 0.0 (ab) | 0.26 ± 0.1 (abc) | 0.2 ± 0.0 (bcd) | 0.16 ± 0.0 (d) | 0.18 ± 0.0 (cd) | † | 0.3 ± 0.1 (abc) | 0.2 ± 0.1 (abcd) | 0.25 ± 0.1 (abcd) | 0.26 ± 0.1 (abc) | 0.2 ± 0.1 (abcd) | 0.23 ± 0.0 (abcd) | 0.2 ± 0.1 (cd) | 0.257 ± 0.1 (abc) |
| % TS | | | | | | | | | | | | | | | | |
| TN | 3 | 3.2 | 3.5 | 3.9 | 5.5 | 6.2 | 6.3 | † | 2.7 | 3.2 | 4.1 | 4.3 | 2.9 | 3 | 3.4 | 3.4 |
| C | 38 | 37 | 37 | 35 | 37 | 37 | 37 | † | 35 | 37 | 38 | 37 | 39 | 37 | 38 | 38 |
| mg/kgTS | | | | | | | | | | | | | | | | |
| Zn | 128 | 161 | 109 | 102 | 129 | 154 | 121 | † | 56 | 75 | 171 | 165 | 72 | 108 | 107 | 102 |
